# Hyperoside ameliorates lupus nephritis by suppressing AKT1-mediated PANoptosis in podocytes: integrating network pharmacology and experimental validation

**DOI:** 10.3389/fphar.2025.1726254

**Published:** 2026-01-07

**Authors:** Lili Cheng, Zhongfu Tang, Ming Li, Chuanbing Huang

**Affiliations:** 1 Department of Rheumatology and Immunology, The First Affiliated Hospital of Anhui University of Chinese Medicine, Hefei, Anhui, China; 2 The First School of Clinical Medicine, Anhui University of Chinese Medicine, Hefei, Anhui, China

**Keywords:** AKT1, hyperoside, lupus nephritis, PANoptosis, podocytes

## Abstract

**Background:**

Lupus nephritis (LN), one of the most common and severe complications of systemic lupus erythematosus (SLE), remains challenging to treat due to its complex pathogenesis. Hyperoside (Hyp), a naturally occurring flavonol glycoside and a key active component in numerous Chinese medicines and herbs, has demonstrated renoprotective effects via multiple signaling pathways, showing promise for LN treatment. However, its underlying mechanisms of renal protection in LN, particularly its regulatory potential on PANoptosis, remain unexplored.

**Objective:**

This study investigated the role of PANoptosis in LN pathogenesis, focusing on protein kinase B (AKT1) -mediated podocyte PANoptosis, to elucidate the therapeutic mechanism of Hyp.

**Methods:**

Potential Hyp targets were predicted using the SwissTargetPrediction database, while LN-related targets were retrieved from the GeneCards database. Overlapping targets were identified as potential key targets, and a Protein-Protein Interaction (PPI) network was constructed to screen core targets. GO and KEGG analyses of these overlapping targets were performed via the DAVID database to predict the mechanisms of Hyp against LN. Molecular docking between Hyp and the core target was conducted using AutoDock (Version 1.5.7) and visualized with PyMOL. Finally, *in vivo* and *in vitro* experiments, including H&E staining, TUNEL staining, immunofluorescence, flow cytometry, Western blotting, immunohistochemistry, and PCR, were performed to assess renal pathology, cell death, and the mRNA/protein expression levels of key targets and PANoptosis markers.

**Results:**

Network pharmacology and molecular docking analyses indicated that AKT1 is a core target shared by Hyp and LN, with Hyp exhibiting stable binding to AKT1. Experimental validation demonstrated that Hyp treatment inhibited podocyte PANoptosis and alleviated renal injury in MRL/lpr mice. Mechanistically, Hyp suppressed PANoptosis by modulating the PI3K/AKT axis. AKT1 overexpression attenuated the therapeutic effects of Hyp, confirming its pivotal role in LN pathogenesis.

**Conclusion:**

This study reveals that AKT1-mediated podocyte PANoptosis is a key mechanism in LN and establishes Hyp as a promising therapeutic agent targeting this pathway. These findings provide a novel and clinically translatable strategy for LN treatment.

## 1 Introduction

Lupus nephritis (LN) ranks as one of the most common and severe complications of systemic lupus erythematosus (SLE), and is a major determinant of long-term patient survival rates and quality of life (Parodis et al., 2025). The pathogenesis of LN is complex, characterized by immune complex deposition, inflammatory infiltration, and progressive renal injury, significantly elevating the risk of end-stage renal failure. If not effectively controlled, LN can rapidly progress to kidney failure, posing a life-threatening risk (Pagkopoulou et al., 2025). Current therapies, such as corticosteroids and immunosuppressants, while offering some benefit, are plagued by issues including suboptimal response rates, significant side effects, and high relapse rates. A proportion of patients do not respond to treatment, developing refractory LN (Izcovich et al., 2025). Consequently, the clinical need for more effective and safer treatments remains largely unmet, underscoring the urgent demand for superior therapeutic strategies.

PANoptosis is a recently identified and distinct form of inflammatory programmed cell death, orchestrated by a multi-protein complex known as the PANoptosome. It integrates key features of pyroptosis, apoptosis, and necroptosis into a unified, synergistic cell death pathway, rather than representing a simple combination of these processes (Chowdhury and Hasan, 2025). Emerging evidence underscores the critical role of PANoptosis in the pathogenesis of LN, positioning it as a central mechanism driving renal injury (Wang et al., 2025). Upon its induction in renal cells—such as glomerular podocytes and tubular epithelial cells—PANoptosis triggers the extensive release of cellular contents and inflammatory mediators, including IL-1β and IL-18. These molecules act as potent “danger signals,” which further recruit and activate immune cells, amplifying local inflammation and tissue damage in a self-sustaining vicious cycle (Guo et al., 2025). Despite its established significance, the upstream drivers and precise molecular mechanisms that trigger PANoptosis in renal podocytes remain incompletely understood and warrant further investigation.

Emerging studies have revealed that PANoptosis, or the coordinated activation of pyroptosis, apoptosis, and necroptosis, plays a critical role in the pathogenesis of systemic autoimmune and inflammatory diseases (Pandian and Kanneganti, 2022; Shi et al., 2023). In systemic lupus erythematosus and related autoimmune conditions, excessive immune activation and sustained inflammatory stress have been shown to concurrently trigger multiple programmed cell death pathways, thereby amplifying tissue damage and disease severity (Funes et al., 2022; Zhao X.Y. et al., 2023). In the kidney, recent evidence indicates that simultaneous activation of pyroptotic, apoptotic, and necroptotic signaling occurs in inflammatory renal disorders, including lupus nephritis, diabetic kidney disease, and ischemic or toxic renal injury. These observations suggest that PANoptosis may represent a convergent mechanism driving renal intrinsic cell injury under chronic inflammatory conditions. However, direct investigation of PANoptosis as an integrated cell death program in lupus nephritis—particularly in glomerular podocytes-and its upstream regulatory mechanisms remain limited.

Hyperoside is a naturally occurring flavonol glycoside with a well-defined chemical structure, identified as quercetin-3-O-galactoside, where quercetin is linked to a galactose moiety. Owing to its immunomodulatory, anti-inflammatory, anti-apoptotic, and mitochondria-protective properties, Hyperoside has shown promise in the treatment of autoimmune diseases (Zhang et al., 2025). Accumulating studies have demonstrated the renoprotective effects of Hyperoside across various disease models, including diabetic nephropathy (Liu et al., 2021), drug-induced nephrotoxicity (Yuan et al., 2023; Elmorsy et al., 2025), and renal ischemia-reperfusion injury (Wu et al., 2019; Li et al., 2023). These beneficial effects are primarily attributed to its antioxidant, anti-inflammatory, and anti-apoptotic activities. By suppressing oxidative stress and inflammatory responses in renal tissues, Hyperoside effectively ameliorates proteinuria, glomerulosclerosis, and tubulointerstitial fibrosis, thereby slowing the progression of kidney injury (Zhang et al., 2016). However, research on Hyperoside in the context of LN remains limited, and its underlying molecular mechanisms are still unclear, which hinders its broader clinical translation. Growing evidence suggests that Hyperoside ameliorates multiple pathological conditions by modulating different programmed cell death pathways—such as pyroptosis, apoptosis, and necroptosis—in various cell types (Zhang et al., 2023; Jin et al., 2025). This body of work implies a potential mechanistic link between the renoprotective effects of Hyperoside and its ability to regulate these critical cell death processes, particularly apoptosis, pyroptosis, and necroptosis.

AKT1, also known as protein kinase B, is a critical serine/threonine kinase that acts as a central node in the PI3K/AKT signaling pathway (Frederick et al., 2022). Upon activation, AKT1 regulates a wide range of cellular processes—including survival, apoptosis, proliferation, growth, metabolism, and inflammatory responses—by phosphorylating numerous downstream substrates (Siddika et al., 2022). Its aberrant activation is recognized as a key factor driving renal inflammation and immune dysregulation in lupus nephritis (LN) (Jiang et al., 2025). Although no study has directly established AKT1 as an upstream regulator of PANoptosis, emerging evidence indicates significant cross-regulation between AKT1 and all three constituent cell death pathways (pyroptosis, apoptosis, and necroptosis) involved in PANoptosis (Larson-Casey et al., 2016; Imamura et al., 2018; Luo et al., 2022). This strongly suggests that AKT1 may serve as an important upstream regulatory node in the PANoptotic process. Collectively, these findings position AKT1 as a potential mediator of PANoptosis in LN pathogenesis and a promising therapeutic target.

In this study, we aimed to address these critical knowledge gaps by investigating the role of PANoptosis in LN and elucidating the therapeutic mechanism of Hyperoside (Hyp). Using an integrated approach combining network pharmacology, molecular docking, *in vivo* experiments in MRL/lpr mice, and *in vitro* cellular models, we explored the involvement of PANoptosis in LN and the pharmacological role of Hyp in modulating this pathway. Furthermore, we identified AKT1 as a central mediator of PANoptosis in LN and a key target underpinning the renoprotective effects of Hyp. By linking a traditional compound with modern mechanistic insights, this study establishes a foundation for the clinical translation of Hyperoside and the development of innovative therapeutic strategies for LN.

## 2 Materials and methods

### 2.1 Network pharmacology and molecular docking

Potential target genes of Hyperoside (Hyp) were predicted using the SwissTargetPrediction database (http://www.swisstargetprediction.ch/) by inputting the chemical structure of Hyp and selecting *Homo sapiens* as the species. Predicted targets with a probability score >0 were retained for subsequent analysis (Daina et al., 2019). Lupus nephritis (LN)–related targets were collected from the GeneCards database (https://www.genecards.org/) using the keyword “lupus nephritis,” and targets with a relevance score above the median value were selected to ensure disease relevance (Stelzer et al., 2016). All retrieved targets were standardized to official gene symbols and deduplicated using the UniProt Knowledgebase (https://www.uniprot.org/). The overlapping targets between Hyp-associated targets and LN-related genes were identified using a Venn diagram, and these shared targets were considered potential therapeutic targets of Hyp in LN, 2025). The overlapping targets between Hyp and LN were identified by constructing a Venn diagram.

**FIGURE 1:**
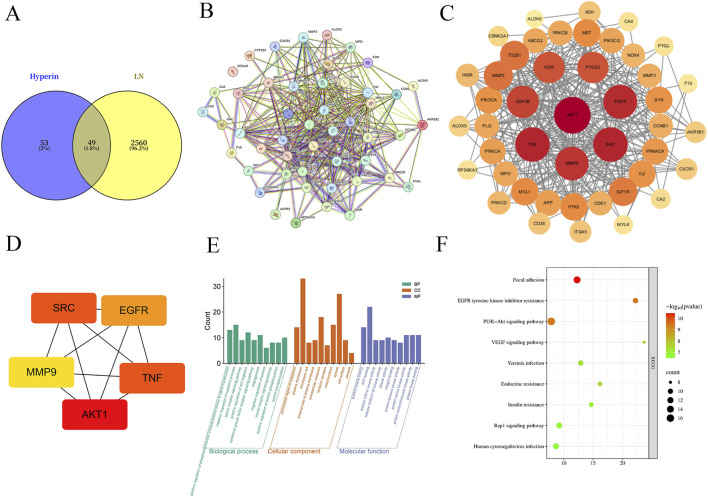
Network pharmacological analysis of Hyperoside and lupus nephritis. **(A)** Venn diagram illustrating the overlapping targets between Hyperoside and LN. **(B)** Global protein-protein interaction (PPI) network of all overlapping targets obtained from STRING. **(C)** Topology-optimized PPI network highlighting key hub targets based on degree centrality. **(D)** Subnetwork of the top 5 core targets. **(E)** Bar plot of Gene Ontology (GO) enrichment analysis for the overlapping targets. **(F)** Bubble plot of KEGG pathway enrichment analysis for the overlapping targets.

Protein–protein interaction (PPI) analysis of the overlapping targets was performed using the STRING database (https://string-db.org/), with the species restricted to *Homo sapiens*. The resulting interaction data were imported into Cytoscape software for network visualization and further analysis. An initial global PPI network including all overlapping targets was constructed to visualize the overall interaction landscape (Figure 1B). Subsequently, topological analysis was performed using the cytoHubba plugin in Cytoscape, and key targets were identified based on degree centrality, which reflects the number of direct interactions with other nodes in the network. A refined PPI network highlighting highly connected hub targets was generated for visualization (Figure 1C). Functional enrichment analysis of the overlapping targets was conducted using the Database for Annotation, Visualization and Integrated Discovery (DAVID, https://david.ncifcrf.gov/) (Chen L. et al., 2017; The UniProt Consortium, 2025).Gene Ontology (GO) analysis was performed to evaluate enriched biological processes, cellular components, and molecular functions, while Kyoto Encyclopedia of Genes and Genomes (KEGG) pathway analysis was applied to identify significantly enriched signaling pathways. Terms with a p value <0.05 were considered statistically significant.

For molecular docking analysis, the three-dimensional structure of Hyperoside was obtained from the Traditional Chinese Medicine Systems Pharmacology (TCMSP) database. The crystal structures of key target proteins were downloaded from the Protein Data Bank (PDB). Prior to docking, proteins were prepared by removing water molecules and adding polar hydrogens using AutoDock Tools. Molecular docking between Hyp and target proteins was performed using AutoDock software (version 1.5.7) with default parameters. Docking conformations were ranked according to binding free energy, and the lowest-energy binding poses were selected for further analysis. Docking results were visualized using PyMOL to illustrate the predicted interaction modes between Hyp and target proteins (Crampon et al., 2022).

### 2.2 Drugs and reagents

Hyperoside (purity >98%, Catalog #: B20631) was purchased from Shanghai Yuanye Bio-Technology Co., Ltd. (Shanghai, China). Prednisone acetate tablets (5 mg/tablet, 100 tablets/bottle, Batch #: H12020689) were obtained from Tianjin Tianyao Pharmaceutical Co., Ltd. (Tianjin, China). The inhibitors VX-765 (HY-13205), Necrostatin-1 (Nec-1, HY-15760), and Z-VAD-FMK (Z-VAD, HY-15760) were sourced from MedChemExpress (MCE, USA).

### 2.3 Cell culture and transient transfection

Mouse podocyte cells (MPC-5) were purchased from iCell Bioscience Inc. (Shanghai, China). Cells were cultured in Dulbecco’s Modified Eagle Medium (DMEM; SH30022.01, Cytiva, USA) supplemented with 10% fetal bovine serum (FBS; 10099-141, Gibco, USA) and 1% penicillin-streptomycin (C0222, Beyotime Biotechnology, China), and maintained at 37 °C in a humidified atmosphere containing 5% CO_2_. Transient transfection of siRNA or overexpression plasmids was performed using Lipofectamine 2000 (2307486, Invitrogen, USA) according to the manufacturer’s instructions. All siRNA and overexpression plasmids were designed and synthesized by General Biol (Anhui, China). Sequence information is provided in Supplementary Material 1.

### 2.4 Preparation of immune complexes (ICs) containing dsDNA

Serum samples were collected from LN patients with high levels of anti-dsDNA antibodies at the Affiliated Hospital of Anhui University of Chinese Medicine. Sera were pooled and stored at −20 °C for subsequent isolation of anti-dsDNA antibodies. For antibody isolation, 1 μg of biotin-dsDNA was incubated with 20 μL of streptavidin magnetic beads in 500 μL of Buffer 1 (10 mM Tris-HCl, 1 M NaCl, 1 mM EDTA) for 30 min at room temperature. After two washes to remove unbound DNA, the biotin-dsDNA-bound beads were resuspended in 400 μL of blocking solution. Then, 100 μL of antiserum containing anti-dsDNA antibodies was added to the beads and incubated overnight at 2 °C–8 °C. Following two washes with 500 μL of Buffer 2 (TBS containing 0.05% Tween-20), the anti-dsDNA antibodies were eluted from the beads using 100 μL of elution buffer (0.1 M glycine solution, pH 2.0) for 10 min at room temperature with intermittent vortexing. Finally, the beads were removed, and 15 μL of neutralization solution (1 M Tris-HCl, pH 8.5) was added to the supernatant to obtain purified anti-dsDNA antibodies. For immune complex (IC) formation, 100 μL of purified anti-dsDNA antibodies were incubated with 10 μg of calf thymus DNA at room temperature for 1 h, and the resulting ICs were stored at −20 °C (Wang et al., 2025). The use of human materials in this study was approved by the Ethics Committee of the Affiliated Hospital of Anhui University of Chinese Medicine (Approval No. 2023AH77).

### 2.5 Cell viability assay (CCK-8)

Cell viability was measured using the Cell Counting Kit-8 (CCK-8; BS350B, Biosharp, Beijing, China) according to the manufacturer’s protocol. Briefly, logarithmically growing cells were digested with trypsin (C0201, Beyotime Biotechnology, China), collected by centrifugation after neutralization, and resuspended to prepare a single-cell suspension. The cell density was adjusted to 4 × 10^4^ cells/mL. After gentle mixing, 100 µL of the cell suspension (containing 8,000 cells) was seeded into each well of a 96-well plate. The peripheral wells were filled with sterile phosphate-buffered saline (PBS; SH30256.01, Cytiva, USA) to minimize evaporation-related artifacts. The plate was incubated overnight at 37 °C, and cell adhesion was observed under an inverted microscope (CKX31, OLYMPUS, Japan). Cells were then treated with various drug concentrations for the indicated durations. Subsequently, 10 µL of CCK-8 reagent was added to each well, followed by further incubation for 1–4 h. The absorbance of each well was measured at 450 nm using a microplate reader, and cell viability was calculated accordingly.

### 2.6 Quantitative real-time PCR (qRT-PCR)

Total RNA was extracted from cells using TRIzol reagent (15596018CN, Invitrogen, USA) following the manufacturer’s instructions. Reverse transcription was performed using the PrimeScript™ RT Reagent Kit with gDNA Eraser (RR047A, TaKaRa, Japan) to synthesize cDNA. For qRT-PCR analysis, a reaction mixture was prepared containing cDNA, Novostart SYBR qPCR SuperMix Plus (E096-01B, Novoprotein, Suzhou, China), and specific primers. Amplification was carried out on a PIKOREAL 96 real-time PCR system (Thermo Scientific, Finland). A Relative Quantification Study was applied for analysis, and the relative mRNA expression levels were calculated using the 2^−ΔΔCT^ method. All primers were designed and synthesized by Sangon Biotech (Shanghai, China). Sequences are listed in Supplementary Material 2.

### 2.7 Western blot analysis

Cells (approximately 1 × 10^5^) or tissue samples (approximately 0.1 g) were lysed in 600 µl of RIPA lysis buffer (P0013B, Beyotime, China) containing 0.6 mM PMSF. The lysates were centrifuged at 12,000 × g for 15 min at 4 °C, and the supernatant containing total protein was collected. Protein samples were separated by SDS-PAGE according to molecular weight and subsequently transferred onto a PVDF membrane (IPVH00010, Millipore, USA) using a wet transfer system. The membrane was then blocked with 5% bovine serum albumin (BSA) or non-fat milk to prevent non-specific antibody binding. After blocking, the membrane was incubated overnight at 4 °C with specific primary antibodies, followed by incubation with corresponding horseradish peroxidase (HRP)-conjugated secondary antibodies. Protein signals were visualized using an enhanced chemiluminescence (ECL) kit (BL520A, Biosharp, China) according to the manufacturer’s instructions. Information regarding all antibodies used is provided in Supplementary Material 3.

### 2.8 Flow cytometry analysis

Cell death was assessed using an Annexin V-FITC/PI Apoptosis Detection Kit (AP101, A20354, LiankeBio, China) following the manufacturer’s protocol. Briefly, harvested cells were washed with cold PBS and resuspended to a density of 1–10 × 10^5^ cells per sample. A 1× binding buffer working solution was prepared by diluting the provided 5× binding buffer with double-distilled water. Cells were resuspended in 500 µL of 1× binding buffer, followed by the addition of 5 µL Annexin V-FITC and 10 µL propidium iodide (PI). After gentle vortexing, the cells were incubated at room temperature for 5 min in the dark. Samples were analyzed immediately using a flow cytometer (CytoFLEX, Beckman Coulter, USA), and the results were processed with appropriate software.

### 2.9 Animal experiments

Female MRL/MpJ and MRL/lpr mice (8 weeks old) were purchased from Shanghai SLAC Laboratory Animal Co., Ltd. (Shanghai, China; License No. SCXK(Hu)2022-0004). All mice were housed under specific pathogen-free (SPF) conditions at the Artificial Intelligence Institute of Hefei Comprehensive National Science Center, with free access to food and water. All animal experiments were reviewed and approved by the Animal Ethics Committee of Anhui University of Chinese Medicine (Approval No. AHUCM-mouse-2024200) and conducted in accordance with the NIH Guide for the Care and Use of Laboratory Animals.

MRL/lpr mice were randomly divided into five groups (n = 6 per group): Model group, Hyp-L (50 mg/kg/day) group, Hyp -M (100 mg/kg/day) group, Hyp-H (200 mg/kg/day) group, and Pred (prednisone 5 mg/kg/day) group. An additional six female MRL/MpJ mice served as the normal control group and received normal saline. Hyperoside and prednisone were dissolved in normal saline (0.9% sodium chloride) to the specified concentrations and administered by oral gavage for 6 weeks. At the study endpoint, mice were euthanized by an intraperitoneal overdose of sodium pentobarbital at 100 mg/kg (solution [30 mg/mL], injection volume [3.3 mL/kg]. Anesthetic depth was verified by loss of righting and pedal-withdrawal reflexes before necropsy. Death was confirmed by absence of heartbeat and respiration with fixed, dilated pupils. The study was approved by the Animal Ethics Committee of Anhui University of Chinese Medicine (No. AHUCM-mouse-2024200)) and followed the AVMA Guidelines for the Euthanasia of Animals (2020) and ARRIVE 2.0 reporting recommendations. Kidney tissue samples and peripheral blood were collected for subsequent analyses, including Western blot, immunohistochemistry (IHC), hematoxylin and eosin (H&E) staining, TUNEL staining, PCR, ELISA, and immunofluorescence (IF).

### 2.10 *In vivo* knockdown and overexpression of AKT1

Adeno-associated virus (AAV) vectors for AKT1 overexpression and knockdown were constructed by Anhui Xinle Biotechnology Co., Ltd. (Hefei, China). For the knockdown experiment, 10-week-old MRL/lpr mice received a tail vein injection (200 µL) of either shRNA negative control AAV (si-NC) or AKT1 knockdown AAV (si-AKT1). Mice were euthanized 8 weeks after AAV injection for further experimental analysis. For the overexpression experiment, 10-week-old MRL/lpr mice were injected via the tail vein (200 µL) with either overexpression negative control AAV (OE-NC) or AKT1 overexpression AAV (OE-AKT1). Four weeks post-injection, mice were treated with Hyperoside or normal saline for an additional 4 weeks before being euthanized for subsequent experiments.

### 2.11 Renal histology and functional analysis

Mouse kidney tissues were fixed in ice-cold 4% paraformaldehyde, embedded in paraffin, and sectioned. Sequential sections (4 µm) were used for the following staining and analyses: Hematoxylin and eosin (H&E) staining was performed to evaluate pathological changes in renal tissues; immunohistochemistry (IHC) was conducted to examine the expression of synaptopodin (21064-1-AP, Proteintech, USA), nephrin (bs-10233R, Bioss, China), podocalyxin (bs-1345R, Bioss, China), and podocin (bs-6597R, Bioss, China); immunofluorescence (IF) staining was used to detect NLRP3 (DF7438, Affinity, UK), RIPK3 (bs-3551R, Bioss, China), and Caspase-9 (bs-0049R, Bioss, China) expression; and a TUNEL assay kit (E-CK-A320, Elabscience, China) was employed to assess cell death. Urinary protein levels were measured using a commercial urine protein assay kit (C035-2-1, Nanjing Jiancheng Bioengineering Institute, Nanjing, China).

### 2.12 Statistical analysis

All data were analyzed using SPSS 26.0 software. Microscopy images were processed and quantified with ImageJ. GraphPad Prism eight was used for generating graphs. For continuous data meeting assumptions of normality and homogeneity of variance, comparisons between two groups were performed using independent samples t-tests, and comparisons among multiple groups were conducted by one-way analysis of variance (ANOVA). A p-value of less than 0.05 was considered statistically significant.

## 3 Results

### 3.1 AKT1 is identified as a potential therapeutic target of hyperoside in LN

Potential target genes of Hyperoside (Hyp) were predicted using the SwissTargetPrediction database, yielding 102 candidates. Meanwhile, 2,609 lupus nephritis (LN)–related targets were retrieved from the GeneCards database. By constructing a Venn diagram, 49 overlapping targets were identified as potential therapeutic targets of Hyp in LN (Figure 1A). These overlapping targets were subsequently subjected to protein–protein interaction (PPI) analysis using the STRING database and visualized in Cytoscape (Figures 1B,C). In the PPI network, node size represents the degree value, with larger nodes indicating a higher number of direct interactions. Topological analysis using the cytoHubba plugin identified the top five hub targets as AKT1, SRC, TNF, EGFR, and MMP9 (Figure 1D). Functional enrichment analysis of the overlapping targets was performed using the DAVID database. Gene Ontology (GO) analysis indicated that the targets were primarily involved in biological processes such as positive regulation of phosphatidylinositol 3-kinase/protein kinase B signal transduction, negative regulation of apoptotic process, and protein phosphorylation (Figure 1E). KEGG pathway analysis revealed significant enrichment in the PI3K-Akt signaling pathway, focal adhesion, and VEGF signaling pathway (Figure 1F).

**FIGURE 2:**
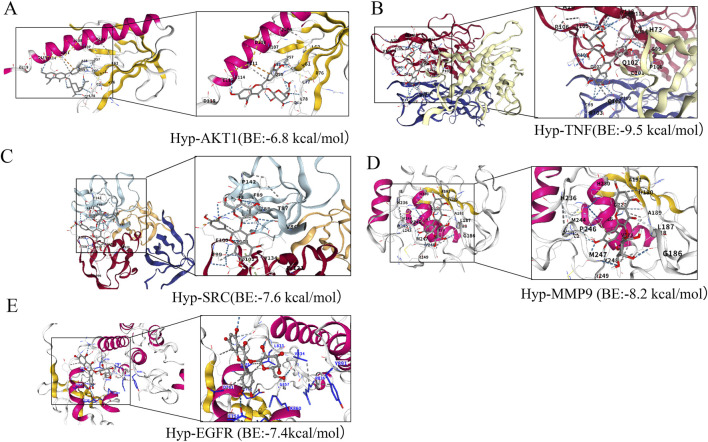
Molecular docking analysis of Hyperoside with core targets. **(A)** Hyp-AKT1 binding mode. **(B)** Hyp-TNF binding mode. **(C)** Hyp-SRC binding mode. **(D)** Hyp-MMP9 binding mode. **(E)** Hyp-EGFR binding mode.

Molecular docking was performed using AutoDock Vina with Hyp as the ligand and the top five hub genes (AKT1, SRC, TNF, EGFR, and MMP9) as receptors. The binding energies for all complexes were lower than −5 kcal/mol (Figure 2), indicating stable binding between Hyp and each target. Based on these results and its highest Degree value in the PPI network, AKT1 was selected as the most promising therapeutic target of Hyp for the treatment of LN.

### 3.2 PANoptosis is associated with the pathology of LN

PANoptosis, as a novel inflammatory cell death pathway, plays a critical role in LN. By mediating the death of renal intrinsic cells and amplifying local inflammation, it drives the initiation, progression, and chronicity of LN. Consequently, targeting the PANoptosis pathway has emerged as a highly promising new strategy for treating LN and other autoimmune diseases (Zhu et al., 2023). In this study, we employed the classic lupus-prone MRL/lpr mouse model and healthy control MRL/MpJ mice to validate the involvement of PANoptosis in LN.

**FIGURE 3:**
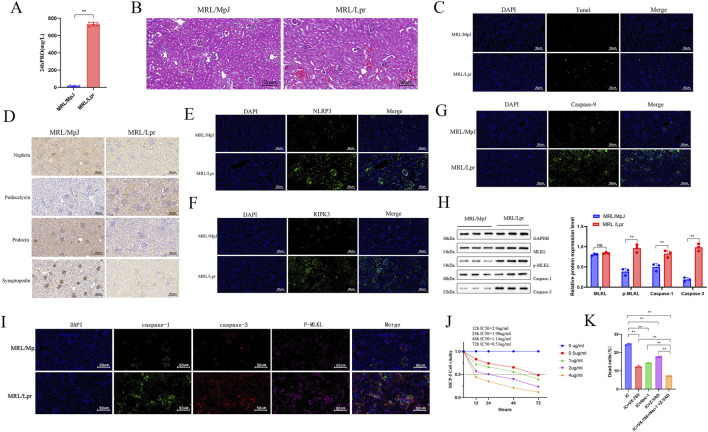
PANoptosis contributes to renal pathology in lupus nephritis. **(A)** Comparison of 24-h urinary protein levels between MRL/MpJ and MRL/lpr mice (n = 6). **(B)** Representative renal histology evaluated by H&E staining (n = 6). **(C)** TUNEL staining showing cell death in glomeruli (n = 6). **(D)** IHC analysis of podocyte markers (Podocalyxin, Nephrin, Synaptopodin, Podocin) in renal tissues. **(E–G)** Immunofluorescence staining of PANoptosis-related markers (NLRP3, RIPK3, Caspase-9) in kidney sections. **(H)** WB analysis of PANoptosis executor proteins (caspase-3, caspase-1, p-MLKL) in renal tissues, with bar graphs showing quantitative results relative to GAPDH (n = 3). **(I)** IF triple-staining of caspase-3, caspase-1, and p-MLKL in mouse kidney tissues. **(J)** Viability of MPC-5 cells treated with ICs at different concentrations and time points, measured by CCK-8 assay. **(K)** Flow cytometric analysis of cell death in MPC5 cells pretreated with VX-765 (10 μM), Nec-1 (20 μM), or Z-VAD (5 μM), followed by IC stimulation for 24 h. Bar graph shows the percentage of dead cells (n = 3). Data are presented as mean ± SD. *p < 0.05, **p < 0.01, ns: not significant.

H&E staining and urinary protein analysis revealed that compared with age-matched MRL/MpJ mice, MRL/lpr mice exhibited increased proteinuria (Figure 3A) and marked renal pathological changes, including significant inflammatory infiltration, glomerular hypertrophy, and glomerular capsular wall hyperplasia (Figure 3B). Consistently, semi-quantitative histopathological scoring demonstrated significantly higher renal activity index (AI) and chronicity index (CI) scores in MRL/lpr mice (Supplementary Figure S2A). TUNEL staining showed multiple positive foci in the glomeruli of MRL/lpr mice (Figure 3C). Given that podocyte injury and death are key mechanisms in LN-related renal dysfunction (Bhargava and Tsokos, 2019; Zhao Q. et al., 2023), we assessed the expression of podocyte death-related proteins by immunohistochemistry. Results showed increased podocalyxin and decreased nephrin, synaptopodin, and podocin in MRL/lpr mice compared with MRL/MpJ mice, indicating podocyte loss in LN kidneys (Figure 3D). Immunofluorescence staining for NLRP3 (He et al., 2016) (a specific marker of pyroptosis), RIPK3 (Morgan and Kim, 2022) (necroptosis), and Caspase-9 (Li et al., 2017) (apoptosis) confirmed the activation of all three death pathways in the kidneys of MRL/lpr mice (Figures 3E–G). Furthermore, Western blot analysis of key PANoptosis protein markers (Shi et al., 2023) showed significantly elevated levels of cleaved caspase-3, caspase-1, and phosphorylated MLKL in MRL/lpr mice compared with healthy controls (Figure 3H). Immunofluorescence triple staining also demonstrated enhanced fluorescence signals for all three markers (Figure 3I).

Previous studies have established that treating podocytes with immune complexes (ICs) containing dsDNA from LN patients can effectively model LN podocyte injury, consistent with the mechanism that abnormal deposition of glomerular ICs underlies podocyte damage in LN (Wang et al., 2025). To further investigate the role of PANoptosis in LN, we stimulated mouse podocytes (MPC5) with dsDNA-containing ICs to establish an *in vitro* model. CCK-8 assay determined the IC50 across different time points, leading to the selection of 2 μg/mL dsDNA ICs treated for 24 h for subsequent experiments (Figure 3J). We pretreated IC-stimulated MPC5 cells individually with VX-765 (a caspase-1 inhibitor) (Wen et al., 2022), Nec-1 (a RIPK1 inhibitor) (Cao and Mu, 2021), or Z-VAD (a pan-caspase inhibitor) (Liao et al., 2022), as well as with their combination. Flow cytometry analysis revealed that each inhibitor significantly reduced the percentage of dead cells. Notably, the combination of VX-765, Nec-1, and Z-VAD suppressed cell death more effectively than any single agent (Figure 3K). Collectively, these results demonstrate that podocyte PANoptosis plays a crucial role in the pathogenesis of LN.

### 3.3 Hyperoside protects podocytes from IC-induced PANoptosis

**FIGURE 4:**
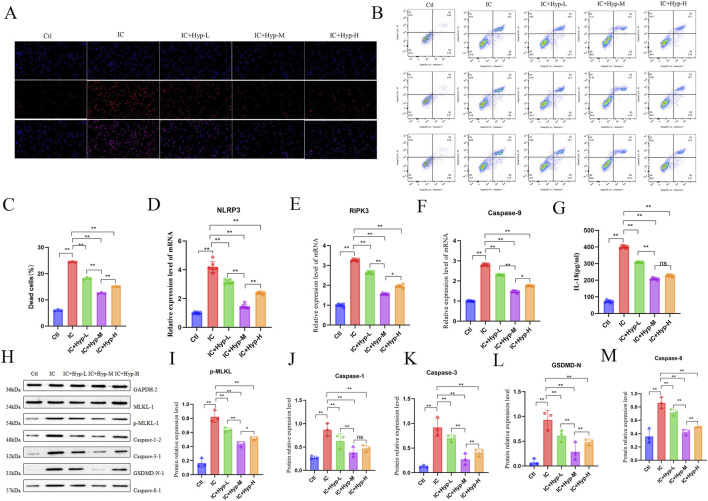
Hyperoside protects podocytes from IC-induced PANoptosis. MPC5 cells were treated with different concentrations of Hyperoside followed by IC stimulation for 24 h **(A)** TUNEL staining showing cell death in MPC5 cells. **(B)** Flow cytometric analysis of apoptosis in MPC5 cells. **(C)** Quantitative analysis of the percentage of dead cells by flow cytometry (n = 3). **(D–F)** mRNA expression levels of PANoptosis-related genes (NLRP3, RIPK3, Caspase-9) measured by qPCR (n = 6). **(G)** IL-18 secretion detected by ELISA (n = 6). **(H–M)** Western blot analysis showing relative protein expression of PANoptosis executors (p-MLKL, Caspase-1, Caspase-3, GSDMD-N, Caspase-8) (n = 3). Data are presented as mean ± SD. *p < 0.05, **p < 0.01, ns: not significant.

As the role of Hyperoside (Hyp) in podocyte PANoptosis remains unclear, this study investigated its effects on IC-induced PANoptosis in MPC5 cells pretreated with different Hyp concentrations. Microscopic imaging (Figure 4A) and flow cytometry analysis (Figures 4B,C) demonstrated that Hyp treatment significantly reduced IC-induced cell death in a dose-dependent manner, with the medium-dose group showing the most pronounced protective effect. Subsequent analysis of PANoptosis-related markers in Hyp-treated MPC5 cells revealed that Hyp significantly suppressed the mRNA expression levels of NLRP3, RIPK3, and Caspase-9 (Figures 4D–F). Consistent with these findings, ELISA showed markedly reduced IL-18 expression following Hyp intervention (Figure 4G). Western blot analysis further confirmed comprehensive suppression of the PANoptosis pathway by Hyp, as evidenced by decreased phosphorylation of MLKL and reduced protein expression of caspase-1, caspase-3, GSDMD-N, and caspase-8 (Figures 4H–M). In addition, analysis of the cleaved (activated) forms of caspase-1, caspase-3, and caspase-8 further demonstrated that Hyp markedly inhibited execution-level activation of PANoptosis in podocytes (Supplementary Figures S1A–D). Moreover, to determine whether IC-induced PANoptosis occurred through the formation of a PANoptosome complex, co-immunoprecipitation (Co-IP) assays were performed. As shown in Supplementary Figures S3A–D, IP of ASC demonstrated the simultaneous association of caspase-8, RIPK3, ZBP1, and NLRP3 in IC-stimulated MPC5 cells. Notably, Hyp treatment markedly reduced the assembly of this multiprotein complex, providing structural evidence that Hyp suppresses PANoptosis by disrupting PANoptosome formation. Collectively, these results indicate that Hyperoside effectively inhibits podocyte PANoptosis.

### 3.4 Hyperoside ameliorates renal pathology and suppresses PANoptosis *in vivo*

**FIGURE 5:**
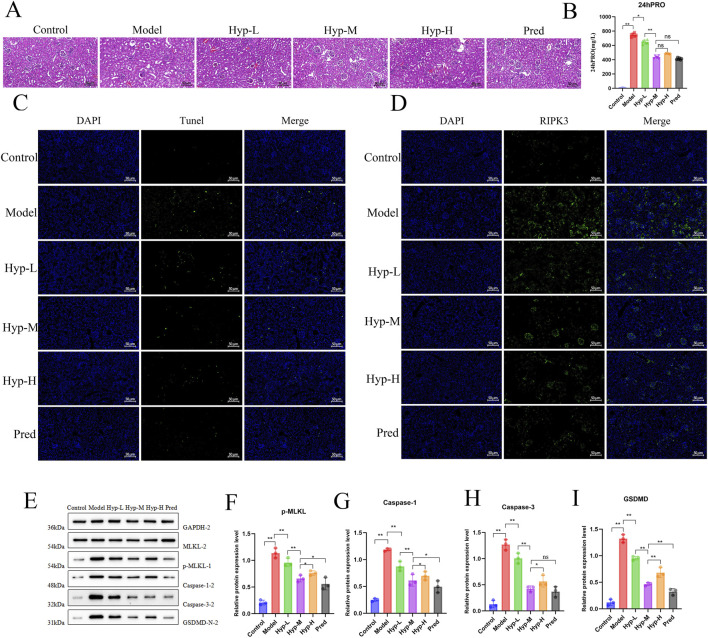
Hyperoside ameliorates renal pathology and suppresses PANoptosis *in vivo*. **(A)** Representative H&E staining showing renal histological changes in MRL/lpr mice treated with different doses of Hyp. **(B)** Comparison of 24-h urinary protein (24hPRO) levels (n = 6). **(C)** TUNEL staining detecting cell death in glomeruli. **(D)** Immunofluorescence staining of PANoptosis-related marker-RIPK3 in kidney sections. **(E–I)** Western blot analysis of PANoptosis executor proteins (p-MLKL, Caspase-1, Caspase-3, GSDMD-N) with quantitative results shown as relative expression levels (n = 3). All data are presented as mean ± SD. *p < 0.05, **p < 0.01, ns: not significant.

To further investigate the effects of Hyp on renal pathology and PANoptosis *in vivo*, MRL/lpr mice were treated with low, medium, and high doses of Hyp for 6 weeks, using prednisone (Pred) as a positive control for LN treatment. Renal and urine samples were collected for subsequent analysis. Compared with the normal control group, model group mice exhibited significant glomerular capsular wall hyperplasia and interstitial inflammatory infiltration. These pathological changes were markedly attenuated in mice treated with Hyp (Hyp-L, Hyp-M, Hyp-H) or Pred (Figure 5A). Consistently, urinary protein levels were significantly lower in Hyp- or Pred-treated mice compared with the model group (Figure 5B). Moreover, semi-quantitative histopathological analysis demonstrated that Hyp treatment significantly reduced both renal AI and CI scores compared with the model group (Supplementary Figures S2B,C). TUNEL staining demonstrated that Hyp treatment effectively reduced the number of dead cells in the glomeruli of MRL/lpr mice (Figure 5C). In addition, immunohistochemical analysis revealed that Hyp treatment decreased podocalyxin expression and increased the expression of nephrin, synaptopodin, and podocin in renal tissues (Supplementary Figure S4A). Meanwhile, immunofluorescence staining showed that intervention with different doses of Hyp or Pred reduced the expression of NLRP3, RIPK3, and Caspase-9 (Figure 5D; Supplementary Figures S4B,C). Western blot analysis indicated significantly decreased protein levels of p-MLKL, caspase-1, caspase-3, and GSDMD-N (Figures 5E–I). These results demonstrate the therapeutic efficacy of Hyp in mitigating renal PANoptosis and improving renal pathology in LN.

### 3.5 Hyperoside suppresses AKT1 expression in podocytes

**FIGURE 6:**
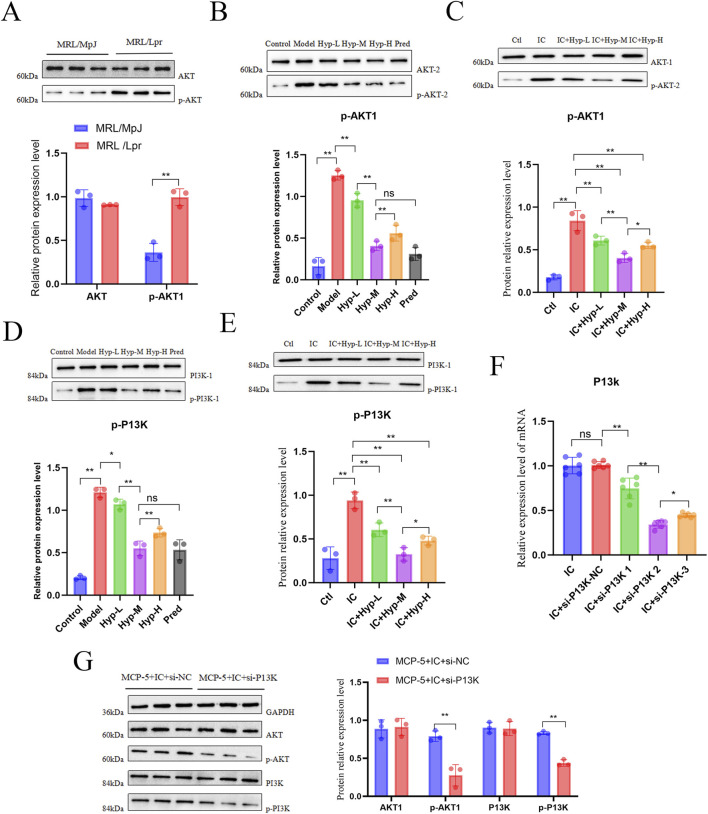
Hyperoside suppresses AKT1 expression in podocytes. **(A)** Relative protein levels of AKT1 and p-AKT1 in renal tissues of MRL/MpJ and MRL/lpr mice (n = 3). **(B)** Effects of different Hyp doses on p-AKT1 levels in renal tissues of MRL/lpr mice (n = 3). **(C)** p-AKT1 expression in MPC-5 cells treated with ICs for 24 h and different Hyp concentrations (n = 3). **(D)** p-PI3K levels in renal tissues of MRL/lpr mice treated with different Hyp doses (n = 3). **(E)** p-PI3K expression in MPC-5 cells treated with ICs for 24 h and different Hyp concentrations (n = 3). **(F)** Screening of optimal si-PI3K concentration by qPCR in MPC5 cells transfected with siNC or different si-PI3K concentrations followed by IC stimulation (n = 6). **(G)** Western blot analysis of AKT1, p-AKT1, PI3K, and p-PI3K protein levels in the indicated groups (n = 3). Data are presented as mean ± SD. *p < 0.05, **p < 0.01, ns: not significant.

To investigate the molecular mechanism by which Hyp regulates PANoptosis, we examined the expression and phosphorylation status of AKT isoforms in renal tissues and podocytes using both *in vivo* and *in vitro* models. Compared with MRL/MpJ mice, renal tissues from MRL/lpr mice exhibited a significant increase in AKT phosphorylation, while total AKT protein levels remained largely unchanged (Figure 6A). Given that AKT2 is the predominant and functionally important AKT isoform in podocytes and the renal cortex, we next assessed both AKT1 and AKT2, as well as their phosphorylated forms, in MRL/lpr mice treated with Hyp. Western blot analysis showed that Hyp treatment dose-dependently reduced AKT phosphorylation, with the medium-dose group showing the most pronounced inhibitory effect. Notably, while phosphorylation of both AKT1 and AKT2 was suppressed, the reduction in p-AKT1 was more prominent (Figure 6B), suggesting a preferential involvement of AKT1 in Hyp-mediated renal protection. To further validate these findings at the cellular level, MPC5 podocytes were stimulated with ICs in the presence or absence of Hyp. IC stimulation markedly increased AKT phosphorylation, whereas Hyp treatment significantly reversed this effect. Consistent with the *in vivo* data, Hyp reduced phosphorylation of both AKT isoforms, with p-AKT1 showing a stronger and dose-dependent decrease (Figure 6C).

We then investigated changes in upstream transcriptional regulators of AKT1 following Hyp treatment. Based on literature review and prior KEGG enrichment analysis, we identified PI3K as a potential upstream regulator of AKT1. Western blot analysis demonstrated that Hyp treatment significantly reduced p-PI3K levels in both MRL/lpr mouse renal tissues and MPC5 cells (Figures 6D,E). To further investigate the role of PI3K in regulating AKT1 expression, we transfected MPC5 cells with PI3K-specific siRNA to knockdown its expression. After initial concentration screening of PI3K siRNA by qPCR (Figure 6F), Western blot analysis confirmed that PI3K knockdown completely abolished p-AKT1 expression (Figure 6G). These results demonstrate that Hyp modulates AKT1 expression by regulating PI3K in podocytes.

### 3.6 AKT1 mediates podocyte PANoptosis and LN progression

**FIGURE 7:**
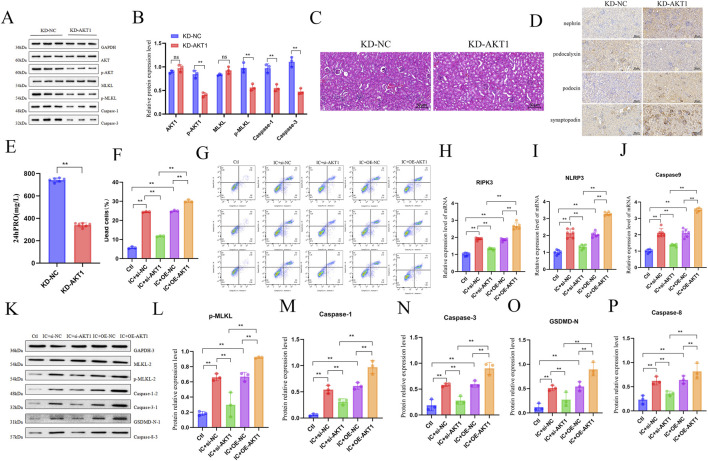
AKT1 mediates podocyte PANoptosis and LN progression. **(A–E)** MRL/lpr mice were injected via tail vein with KD-NC or KD-AKT1 AAV and maintained for 6 weeks (n = 6). **(A,B)** Western blot analysis of AKT1 and PANoptosis markers (p-MLKL, Caspase-1, Caspase-3) in renal tissues (n = 3). **(C)** Renal pathology assessed by H&E staining. **(D)** Immunohistochemical staining of podocyte-associated proteins (Podocalyxin, Nephrin, Synaptopodin, Podocin). **(E)** Comparison of 24 h urinary protein levels between the two groups. **(F–P)** MPC-5 cells were transfected with Si-NC, Si-AKT1, OE-NC, or OE-AKT1, followed by IC treatment for 24 h. **(F)** Percentage of dead cells measured by flow cytometry (n = 6). **(G)** Representative flow cytometry plots showing apoptosis. **(H–J)** mRNA expression levels of PANoptosis-related genes (NLRP3, RIPK3, Caspase-9) detected by qPCR (n = 6). **(K–P)** Western blot analysis of PANoptosis executor proteins (p-MLKL, Caspase-1, Caspase-3, GSDMD-N, Caspase-8) in MPC5 cells (n = 3). Data are presented as mean ± SD. *p < 0.05, **p < 0.01, ns: not significant.

To investigate the role of AKT1 in LN pathology, we delivered renal-targeting AAV vectors carrying AKT1-specific shRNA (KD-AKT1) or control shRNA (KD-NC) to MRL/lpr mice via tail vein injection to achieve kidney-specific AKT1 knockdown. Six weeks post-injection, renal tissues and urine samples were collected for pathological evaluation. Western blot analysis confirmed efficient AKT1 knockdown in the KD-AKT1 group, accompanied by reduced levels of cleaved caspase-3, caspase-1, and phosphorylated MLKL (Figures 7A,B). H&E staining revealed that KD-AKT1 mice exhibited significant amelioration of lupus-like glomerular pathology and interstitial inflammatory cell infiltration compared to KD-NC controls (Figure 7C). Consistently, semi-quantitative histopathological scoring showed significantly reduced renal AI and CI scores in KD-AKT1 mice (Supplementary Figure S2D). Immunohistochemistry showed increased podocalyxin and decreased nephrin, synaptopodin, and podocin expression in KD-AKT1 mouse kidneys (Figure 7D). Furthermore, 24-h urinary protein levels were substantially lower in KD-AKT1 mice than in KD-NC mice (Figure 7E). These results demonstrate the critical role of AKT1 in promoting LN progression. We next examined the role of AKT1 in regulating podocyte PANoptosis *in vitro*. MPC5 cells were transfected with AKT1-specific siRNA or pcDNA3.1-AKT1 to evaluate their effects on IC-induced PANoptosis. Flow cytometry analysis showed that AKT1 knockdown effectively protected podocytes from IC-induced cell death, whereas AKT1 overexpression exacerbated cell death (Figures 7F,G). Additionally, qPCR analysis revealed that AKT1 overexpression upregulated the mRNA expression of RIPK3, NLRP3, and Caspase-9 (Figures 7H–J). Western blot analysis demonstrated that AKT1 knockdown significantly suppressed the activation of p-MLKL, caspase-1, caspase-3, GSDMD-N, and caspase-8 in MPC5 cells, while AKT1 overexpression produced the opposite effects (Figures 7K–P). In addition, analysis of the cleaved forms of caspase-1, caspase-3, and caspase-8 further confirmed that AKT1 regulates execution-level activation of PANoptosis in podocytes (Supplementary Figures S1E–H). In summary, these findings establish AKT1 as a key regulator of podocyte PANoptosis and LN progression.

### 3.7 Hyperoside attenuates AKT1-mediated PANoptosis in LN

**FIGURE 8:**
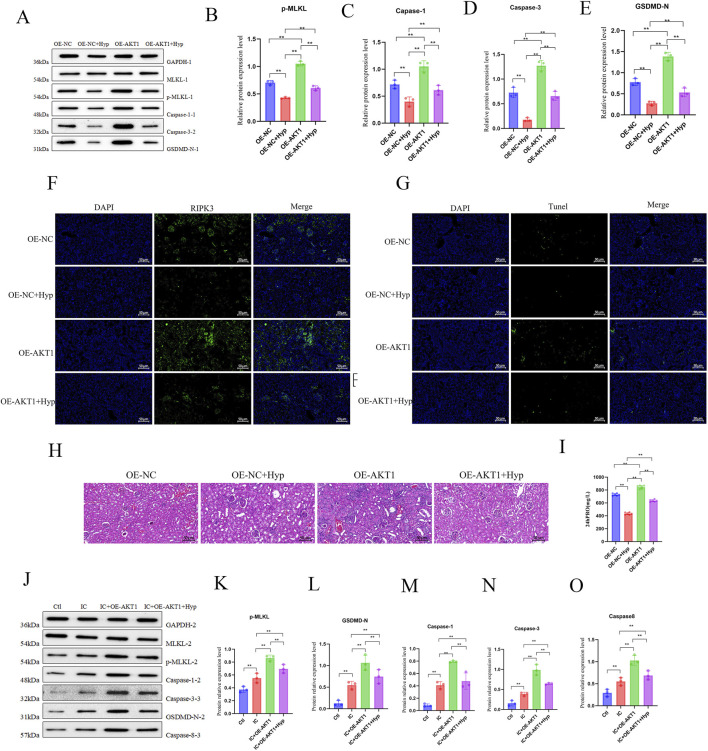
Hyperoside alleviates AKT1-mediated PANoptosis in lupus nephritis. MRL/lpr mice were injected via tail vein with OE-NC or OE-AKT1 AAV. After 4 weeks, mice were treated daily with normal saline (NS) or Hyperoside (Hyp, 100 mg/kg) by oral gavage for 4 weeks (n = 6). **(A–E)** Western blot analysis of PANoptosis-related proteins (p-MLKL, Caspase-1, Caspase-3, GSDMD-N, Caspase-8) in renal tissues (n = 3). **(F)** Immunofluorescence staining of RIPK3 in kidney sections. **(G)** TUNEL assay detecting cell death in renal tissues. **(H)** Renal pathology evaluated by H&E staining. **(I)** Comparison of 24 h urinary protein levels among groups. **(J–O)** MPC-5 cells stably overexpressing AKT1 or control vector were treated with ICs for 24 h, followed by NS or Hyp treatment. Western blot analysis of PANoptosis executor proteins (p-MLKL, Caspase-1, Caspase-3, GSDMD-N, Caspase-8) in MPC5 cells (n = 3). Data are presented as mean ± SD. *p < 0.05, **p < 0.01, ns: not significant.

To determine whether Hyperoside (Hyp) ameliorates renal injury and podocyte PANoptosis through AKT1, MRL/lpr mice received renal-targeting AAV vectors carrying AKT1 overexpression construct (OE-AKT1) or negative control vector (OE-NC) via tail vein injection. Starting from the fourth week post-injection, mice were treated daily with Hyp or normal saline by oral gavage for 4 weeks. Renal tissues and urine samples were collected for analysis after euthanasia. Western blot analysis demonstrated that Hyp effectively suppressed the activation of caspase-1, caspase-3, p-MLKL, and GSDMD-N, while AKT1 overexpression attenuated these inhibitory effects of Hyp (Figures 8A–E). Furthermore, Hyp treatment counteracted the effects of AKT1 overexpression on the levels of NLRP3, RIPK3, and caspase-9 (Figure 8F; Supplementary Figures S5A,B). Immunohistochemistry revealed that Hyp reduced the AKT1 overexpression-induced upregulation of podocalyxin and restored the expression of nephrin, synaptopodin, and podocin (Supplementary Figure S5C). TUNEL staining indicated that Hyp treatment decreased cell death exacerbated by AKT1 overexpression (Figure 8G). Additionally, Hyp alleviated AKT1 overexpression-aggravated renal inflammation, lupus-like pathological changes (Figure 8H), and proteinuria (Figure 8I) in MRL/lpr mice. Consistently, semi-quantitative histopathological analysis showed that Hyp treatment significantly reduced both renal AI and CI scores in AKT1-overexpressing mice (Supplementary Figures S2E,F). Finally, we established stable AKT1-overexpressing and control MPC5 cell lines treated with ICs and/or Hyp to verify the crucial role of AKT1 in mediating Hyp’s regulation of PANoptosis. Hyp significantly reduced the percentage of dead cells in IC-stimulated cells (Supplementary Figure S5D) and inhibited PANoptosis pathway activation, effects that were markedly diminished in AKT1-overexpressing MPC5 cells (Figures 8J–O; Supplementary Figures S5E–G). Consistently, analysis of the cleaved forms of caspase-1, caspase-3, and caspase-8 further confirmed that AKT1 overexpression attenuated Hyp-mediated suppression of execution-level PANoptosis (Supplementary Figures S1I–L). In conclusion, these results demonstrate that AKT1 serves as a key target of Hyp, through which Hyp exerts its therapeutic effects in LN by suppressing podocyte PANoptosis and improving renal pathology and function.

## 4 Discussion

In this study, we elucidated the pharmacological mechanism by which Hyp) protects glomerular cells from PANoptosis in LN. Through an integrated approach combining network pharmacology, molecular docking, *in vivo* experiments in MRL/lpr mice, and *in vitro* cellular models, we demonstrated that Hyp alleviates renal injury in LN by inhibiting the phosphorylation of AKT1, a key regulator of PANoptosis. These findings not only deepen our understanding of LN pathogenesis but also provide a mechanistic foundation for the clinical application of Hyp in LN treatment.

The roles of programmed cell death pathways—including pyroptosis, necroptosis, and apoptosis—in LN pathogenesis have been extensively studied and recognized (Wang et al., 2017). However, the involvement of PANoptosis, a comprehensive cell death pathway integrating features of all three death modalities, remains insufficiently investigated in LN. In this study, our *in vivo* and *in vitro* experiments confirmed the simultaneous activation of pyroptosis, necroptosis, and apoptosis in glomerular cells, indicating that PANoptosis plays a central role in LN pathogenesis. This finding opens new avenues for therapeutic intervention by targeting PANoptosis in LN.

Oxidative stress and inflammation are tightly interconnected pathological processes in lupus nephritis and mutually reinforce each other to exacerbate renal injury. In podocytes, excessive reactive oxygen species (ROS) generated in response to immune complex deposition and inflammatory cytokines not only amplify inflammatory signaling but also serve as a common upstream trigger for multiple forms of programmed cell death (Halfon et al., 2024). Accumulating evidence indicates that oxidative stress can promote NLRP3 inflammasome activation leading to pyroptosis, induce mitochondrial dysfunction–dependent intrinsic apoptosis, and facilitate RIPK3/MLKL-mediated necroptosis. Importantly, these death pathways share overlapping upstream stress signals and downstream molecular mediators, enabling their simultaneous and cooperative activation rather than functioning as isolated processes (Iba et al., 2025). PANoptosis has therefore been proposed as an integrated programmed cell death pathway that reflects the convergence of pyroptosis, apoptosis, and necroptosis under conditions of severe inflammatory and oxidative stress (Liu et al., 2024). In the context of lupus nephritis, sustained oxidative stress within the glomerular microenvironment may drive this convergence in podocytes. Our findings of concurrent activation of all three death modalities in podocytes both *in vivo* and *in vitro* are consistent with this integrative PANoptosis model.

Protein kinase B (AKT1), a serine/threonine kinase, regulates fundamental cellular processes including cell survival, proliferation, pyroptosis, apoptosis, and necroptosis. AKT1 activity is controlled by two regulatory phosphorylation sites (Thr308 and Ser473), which stimulate downstream signaling cascades through phosphorylation of numerous target proteins (Siddika et al., 2024), positioning AKT1 as a core molecular regulator of PANoptosis and a pivotal player in LN pathogenesis. Notably, dysregulated AKT1 signaling has been implicated in oxidative stress responses, inflammasome activation, mitochondrial homeostasis, and cell fate determination, positioning AKT1 as a pivotal molecular node linking inflammation, oxidative stress, and PANoptosis (Xu et al., 2025). In this study, we observed significantly elevated levels of both AKT1 and p-AKT1 in renal tissues from LN patients and mouse models, with this increase positively correlating with PANoptosis pathway activation. Importantly, we demonstrated that AKT1 overexpression exacerbated PANoptosis in glomerular cells, thereby aggravating renal pathology—manifested as enhanced renal inflammation, aggravated lupus-like pathological changes, and increased proteinuria. Conversely, AKT1 knockdown using specific siRNA conferred resistance to IC-induced PANoptosis in podocytes, providing compelling evidence for AKT1 as a potential therapeutic target in LN. Although previous studies have delineated the role of AKT1 in SLE and LN, our research is the first to reveal its crucial function in renal cells, establishing AKT1 as a promising therapeutic target for LN.

Beyond its upstream positioning in the PI3K/AKT pathway, AKT1 exerts its regulatory effects through a range of downstream effectors that collectively influence inflammation and multiple forms of programmed cell death. Activation of AKT1 promotes mTOR signaling, which has been implicated in the regulation of inflammatory responses, cellular metabolism, and inflammasome-related signaling, potentially modulating pyroptotic activity under chronic inflammatory conditions (Weichhart et al., 2015; Rodriguez-Colman et al., 2024). In parallel, AKT1 negatively regulates FOXO family transcription factors, key mediators of oxidative stress responses and transcriptional control of pro-apoptotic genes, thereby influencing mitochondrial apoptosis.

AKT1 also directly phosphorylates and inactivates the pro-apoptotic protein BAD, providing a mechanistic link between AKT signaling and intrinsic apoptotic pathways. Moreover, emerging evidence suggests functional crosstalk between AKT signaling and RIPK1-associated cell death pathways, implicating AKT1 in the regulation of necroptosis. Through coordinated modulation of these downstream targets, dysregulated AKT1 activity may simultaneously lower the threshold for pyroptosis, apoptosis, and necroptosis, thereby facilitating the integrated execution of PANoptosis in podocytes under sustained inflammatory and oxidative stress conditions.

Hyperoside, a flavonoid glycoside extracted from plants such as *Hypericum perforatum* (Wang et al., 2023), has been extensively studied for its remarkable anti-inflammatory and antioxidant activities. It has been widely used in China to treat various kidney diseases, including chronic kidney disease (An et al., 2017; Chen Z. et al., 2017; Zhou et al., 2021). Previous research has primarily focused on the modulatory effects of Hyperoside on immune cell function, whereas its direct protective role on renal intrinsic cells, particularly in LN, remains insufficiently explored. In this study, we demonstrated that Hyperoside effectively blocks PANoptosis pathway activation by inhibiting AKT1 phosphorylation in podocytes, thereby protecting glomerular cells in both *in vivo* and *in vitro* LN models. Furthermore, AKT1 overexpression attenuated the protective effects of Hyperoside, confirming that its renoprotective action is mediated through the suppression of AKT1-driven PANoptosis. Our study also identified PI3K as a potential upstream regulator of AKT1. This finding aligns with previous studies demonstrating that bioactive components derived from traditional Chinese medicine can modulate disease processes through the PI3K/AKT signaling pathway (Liu et al., 2025). This mechanism not only explains the significant efficacy of Hyperoside in improving renal pathology and function but also establishes a scientific basis for its clinical application in LN treatment.

Accumulating evidence indicates that Hyperoside and its bioactive components exhibit therapeutic potential for various diseases by modulating key cell death pathways, including pyroptosis, apoptosis, and necroptosis. For instance, studies have shown that Hyperoside prevents cadmium-induced renal inflammation by suppressing ROS/MAPK/NF-κB-mediated NLRP3 inflammasome activation (Li et al., 2023). Similarly, Hyperoside has been demonstrated to protect against diabetic kidney disease through regulation of the ROS-ERK signaling pathway and pyroptosis (Zhang et al., 2023). Additionally, research has revealed that Hyperoside induces apoptosis in breast cancer cells via the ROS-mediated NF-κB signaling pathway (Qiu et al., 2019). Building upon these findings, our study innovatively reveals that Hyperoside may regulate a more complex programmed cell death pathway—PANoptosis—and identifies AKT1 as its key regulatory target in LN. The diverse bioactive compounds in Hyperoside and their broad regulatory targets highlight its therapeutic potential for various kidney diseases.

Accumulating evidence indicates that Hyperoside regulates multiple programmed cell death pathways in a context-dependent manner. Previous studies have shown that Hyperoside suppresses pyroptosis by attenuating oxidative stress and inflammatory signaling in renal injury models, whereas it induces apoptosis in certain cancer cells through ROS-related pathways. These seemingly divergent effects likely reflect differences in cellular context and stress intensity rather than contradictory mechanisms (Li et al., 2023; Zhang et al., 2023).

In podocytes exposed to sustained inflammatory and oxidative stress, as occurs in lupus nephritis, we propose that Hyperoside attenuates excessive stress signaling and interrupts the convergence of pyroptotic, apoptotic, and necroptotic pathways into PANoptosis. Inhibition of AKT-dependent signaling may therefore represent a unifying upstream mechanism by which Hyperoside restrains oxidative stress–associated inflammation and suppresses PANoptosis, ultimately preserving podocyte integrity and renal function.

Despite these significant findings, our study has several limitations that warrant further investigation. First, Hyperoside is a flavonoid glycoside with complex *in vivo* metabolites. Although our molecular docking results confirmed that Hyperoside directly binds to AKT1, future studies should systematically characterize the active metabolites of Hyperoside in renal tissues and their regulatory effects on the AKT1-PANoptosis axis. Structural biology approaches, such as cryo-electron microscopy, should be employed to precisely resolve the molecular conformation of Hyperoside bound to AKT1. Furthermore, while our research indicates that Hyperoside suppresses podocyte PANoptosis by inhibiting AKT1 signaling, the complex downstream regulatory network involving multiple substrates such as mTOR, FOXO, BAD, and RIPK1 requires further exploration. Elucidating these mechanisms will provide a more comprehensive understanding of the renoprotective effects of Hyperoside and facilitate the development of novel targeted therapies for diseases such as LN.

## 5 Conclusion

In summary, our study unveils a critical pathogenic mechanism in lupus nephritis by establishing AKT1-mediated podocyte PANoptosis as a key driver of renal injury. We further identify a novel pharmacological mechanism through which hyperoside exerts renoprotective effects by suppressing AKT1 phosphorylation and PANoptosis in glomerular cells. This discovery provides new therapeutic perspectives for SLE-induced renal damage, demonstrating through integrated *in vivo* and *in vitro* evidence that hyperoside not only regulates the expression of critical podocyte structural proteins but also effectively inhibits the interconnected network of inflammation and programmed cell death pathways. The multi-target and multi-level interventional advantages of hyperoside highlighted in our work offer fresh insights into its mechanism of action in SLE-related renal injury and establish an experimental foundation for developing therapeutics targeting the AKT1 signaling pathway.

## Data Availability

The original contributions presented in the study are included in the article/Supplementary Material, further inquiries can be directed to the corresponding author.

## Supplementary material

SUPPLEMENTARY FIGURE S1 MPC-5 podocytes were stimulated with ICs and treated with low-, medium-, or high-dose Hyperoside (Hyp-L, Hyp-M, Hyp-H), or subjected to AKT1 knockdown or overexpression as indicated. **(A–D)** Western blot analysis of cleaved-caspase-1, cleaved-caspase-3, and cleaved-caspase-8 in MPC-5 cells under control conditions (Ctl), IC stimulation, and IC stimulation followed by Hyp treatment. GAPDH was used as a loading control. **(E–H)** Western blot analysis of cleaved-caspase-1, cleaved-caspase-3, and cleaved-caspase-8 in MPC-5 cells transfected with control siRNA (si-NC), AKT1 siRNA (si-AKT1), control overexpression vector (OE-NC), or AKT1 overexpression vector (OE-AKT1) under IC stimulation. **(I–L)** MPC-5 cells stably overexpressing AKT1 or control vector were treated with ICs for 24 hours, followed by normal saline (NS) or Hyperoside (Hyp) treatment. Western blot analysis of cleaved-caspase-1, cleaved-caspase-3, and cleaved-caspase-8 was performed. Quantitative analysis of protein expression levels is shown for each corresponding panel. Data are presented as mean ± SD (n = 3). **p* < 0.05, ***p* < 0.01, ns: not significant.

SUPPLEMENTARY FIGURE S2 Semi-quantitative histopathological scoring of renal injury in lupus nephritis. **(A)** Comparison of activity index (AI) and chronicity index (CI) scores between MRL/MpJ and MRL/lpr mice. **(B,C)** Semi-quantitative analysis of renal pathological injury based on AI **(B)** and CI **(C)** scores in control mice, MRL/lpr model mice, and MRL/lpr mice treated with low-, medium-, or high-dose Hyperoside (Hyp-L, Hyp-M, Hyp-H) or Pred. **(D)** Comparison of AI and CI scores in MRL/lpr mice receiving kidney-targeted AAV-mediated AKT1 knockdown (KD-AKT1) or negative control (KD-NC). **(E,F)** Semi-quantitative analysis of AI **(E)** and CI **(F)** scores in MRL/lpr mice overexpressing AKT1 (OE-AKT1) or control vector (OE-NC), with or without Hyperoside treatment. Renal histopathological scores were evaluated based on hematoxylin and eosin (H&E)-stained kidney sections according to established scoring criteria. Data are presented as mean ± SD (n = 3). **p* < 0.05, ***p* < 0.01, ns: not significant.

SUPPLEMENTARY FIGURE S3 MPC-5 podocytes were stimulated with immune complexes (ICs) with or without low-dose Hyperoside (L-Hyp). Co-immunoprecipitation (Co-IP) assays were performed to analyze protein complex assembly. **(A)** Cell lysates were immunoprecipitated with anti-ASC or anti-caspase-8 antibodies, followed by immunoblotting for ASC and caspase-8. Input and IgG were used as controls. **(B)** Co-IP analysis of the interaction between ASC and RIPK3 using anti-ASC or anti-RIPK3 antibodies. **(C)** Co-IP analysis of the interaction between ASC and ZBP1 using anti-ASC or anti-ZBP1 antibodies. **(D)** Co-IP analysis of the interaction between ASC and NLRP3 using anti-ASC or anti-NLRP3 antibodies. These data show that caspase-8, RIPK3, ZBP1, and NLRP3 are detected within ASC-containing protein complexes upon IC stimulation, and that Hyperoside treatment reduces the assembly of this complex.

SUPPLEMENTARY FIGURE S4
**(A)** Immunohistochemical analysis of podocyte-associated proteins (Podocalyxin, Nephrin, Synaptopodin, Podocin) in renal tissues. **(B,C)** Immunofluorescence staining of PANoptosis-related markers (NLRP3 and Caspase-9) in kidney sections.

SUPPLEMENTARY FIGURE S5
**(A,B)** Immunofluorescence staining of NLRP3 and Caspase-9 in kidney sections. **(C)** Immunohistochemical analysis of podocyte-associated proteins (Podocalyxin, Nephrin, Synaptopodin, Podocin). **(D–G)** MPC-5 cells stably overexpressing AKT1 or control vector were treated with ICs for 24 hours, followed by NS or Hyp treatment. **(D)** Percentage of dead cells measured by flow cytometry (n=3). **(E–G)** mRNA expression levels of PANoptosis-related genes (NLRP3, RIPK3, Caspase-9) detected by qRT-PCR (n=3). All data are presented as mean ± SD. **p* < 0.05, ***p* < 0.01, ns, not significant.
