# Supplementary figures and images for: Hyperoside ameliorates lupus nephritis by suppressing AKT1-mediated PANoptosis in podocytes: integrating network pharmacology and experimental validation
Source: Front Pharmacol. 2026 Jan 7;16:1726254. doi: 10.3389/fphar.2025.1726254 (PMC12819326; doi:10.3389/fphar.2025.1726254)

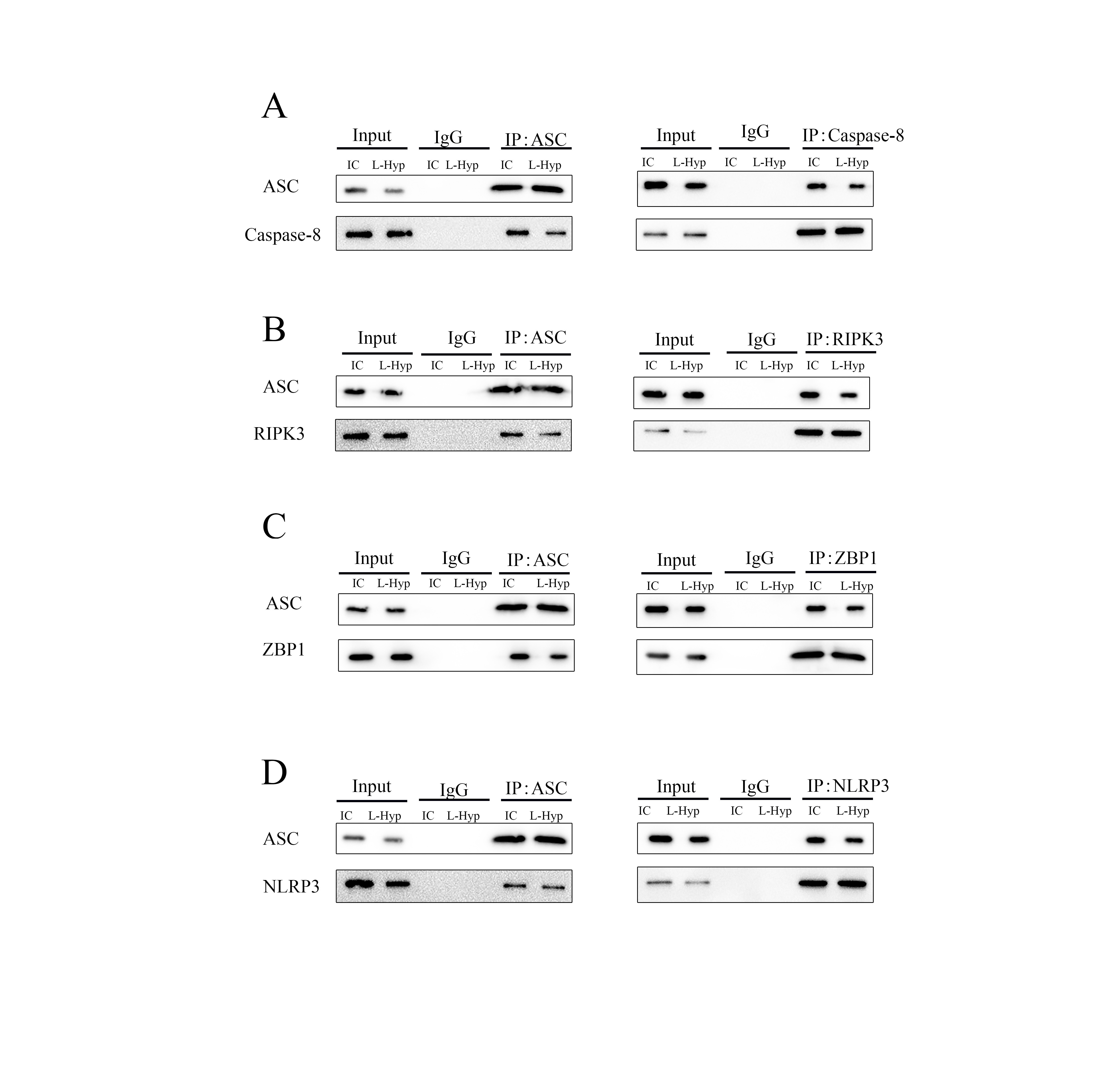

Supplement: Supplementary file 1 [file Image3.jpeg]

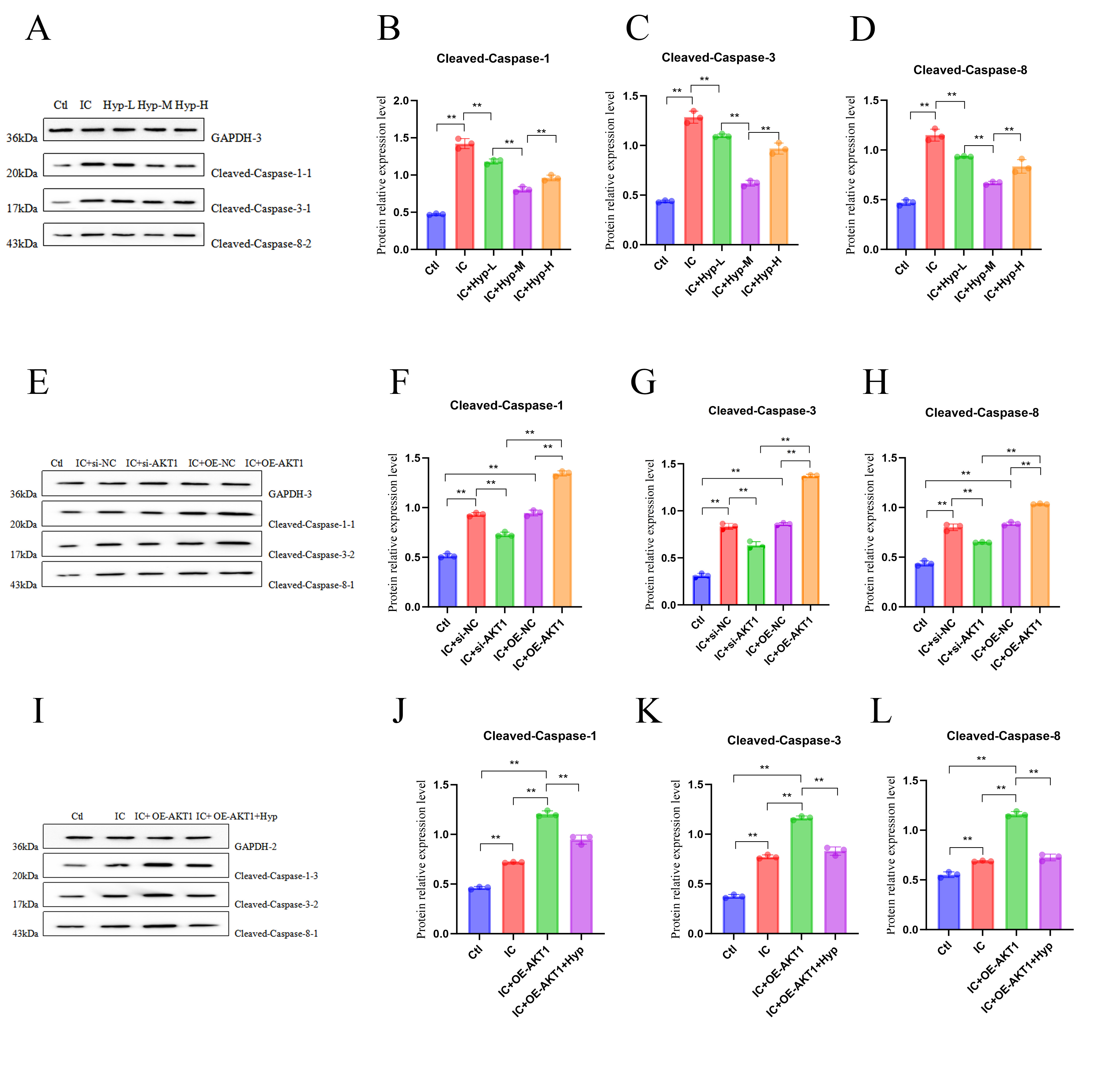

Supplement: Supplementary file 3 [file Image1.jpeg]

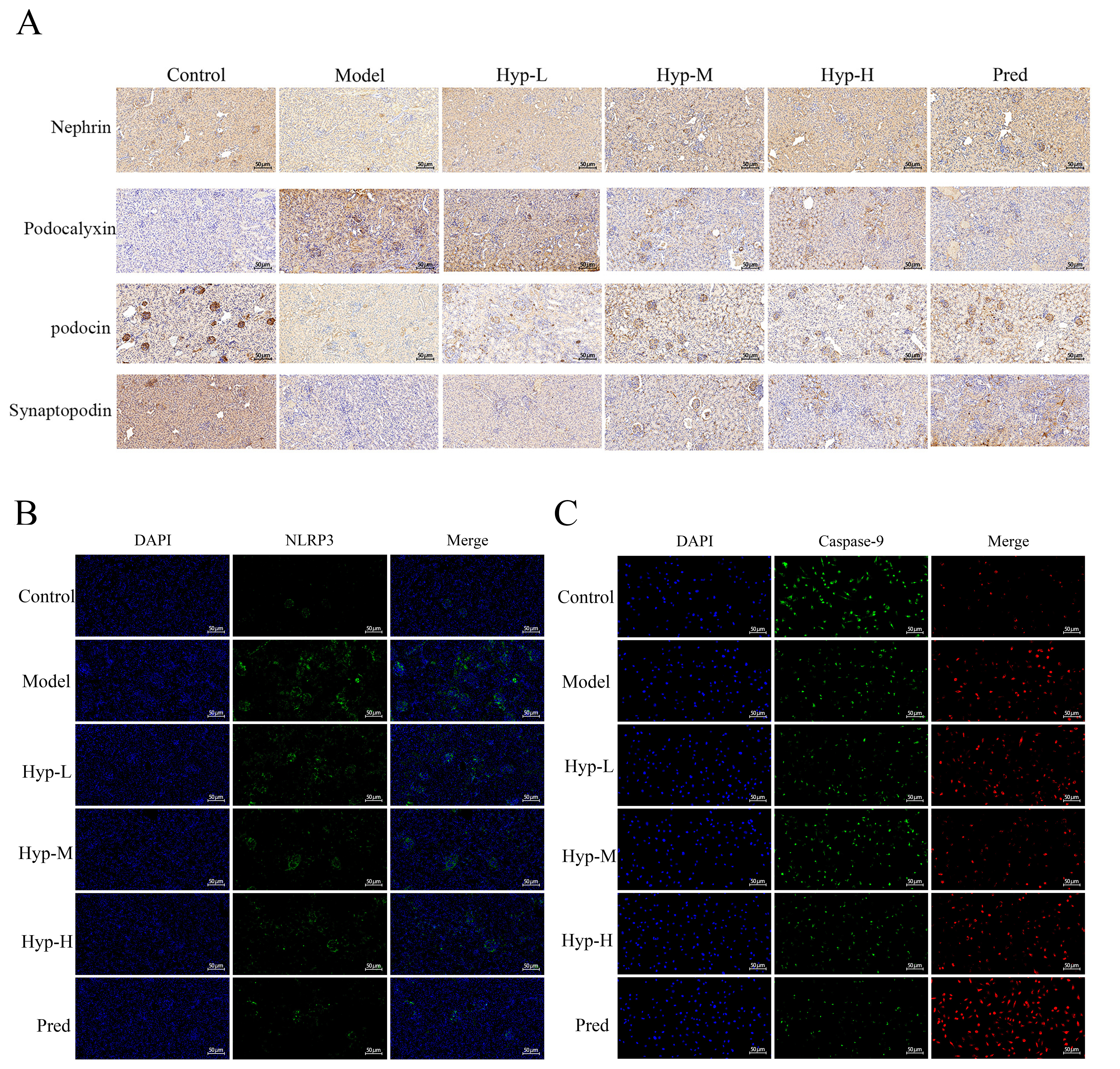

Supplement: Supplementary file 4 [file Image4.jpeg]

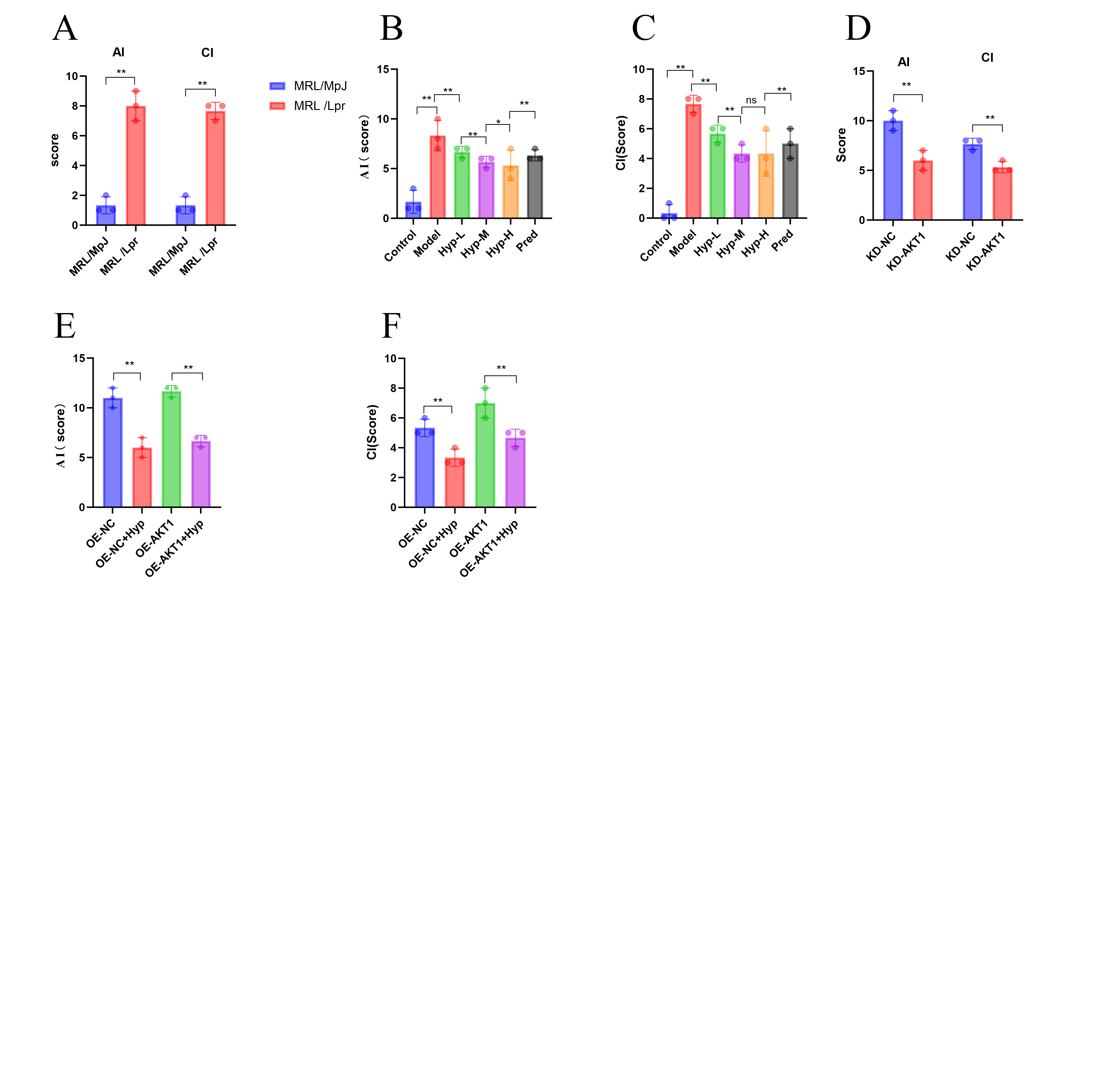

Supplement: Supplementary file 5 [file Image2.jpeg]

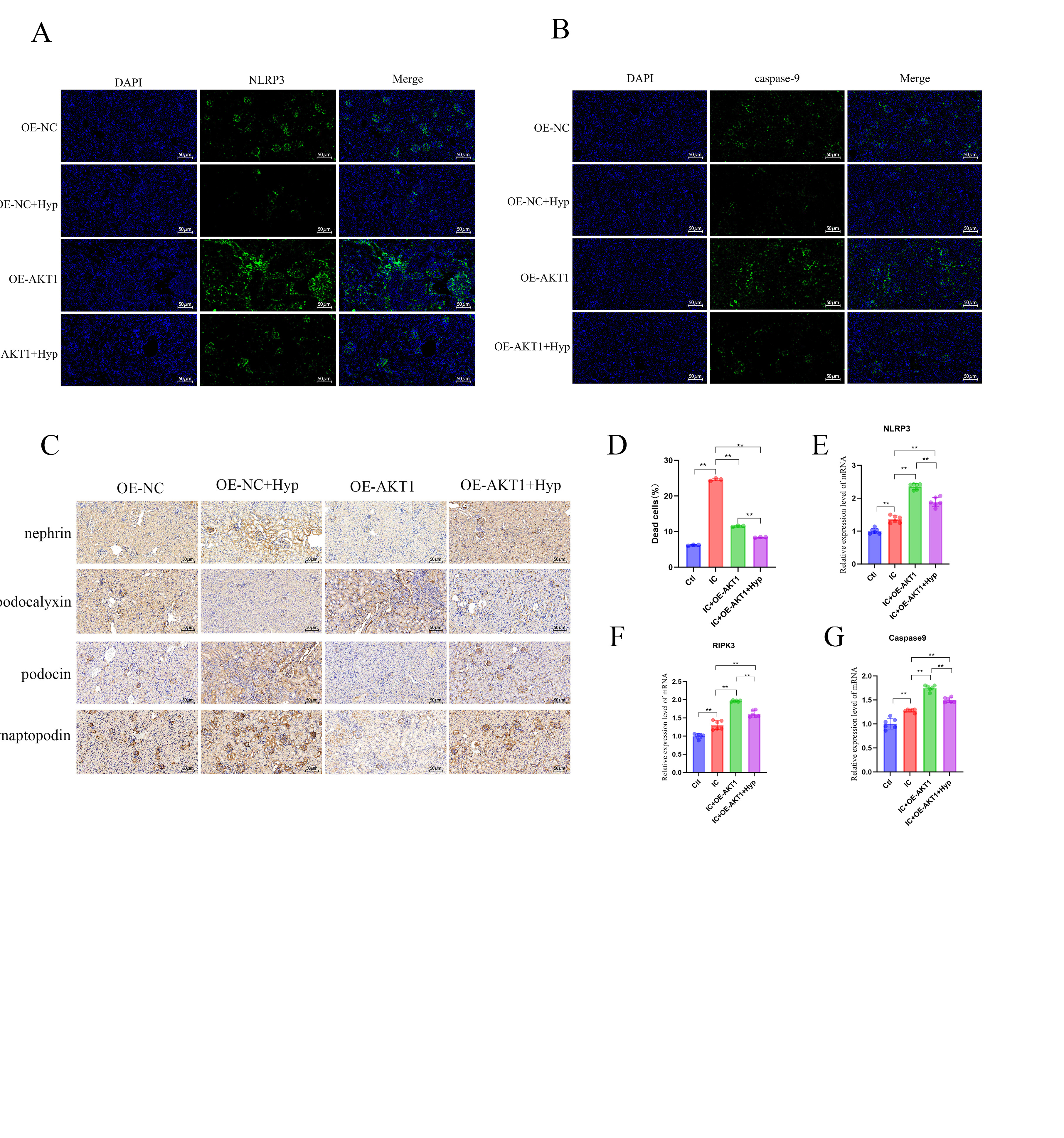

Supplement: Supplementary file 6 [file Image5.jpeg]
