# Supplementary material for: Hyperoside ameliorates lupus nephritis by suppressing AKT1-mediated PANoptosis in podocytes: integrating network pharmacology and experimental validation
Source: Front Pharmacol. 2026 Jan 7;16:1726254. doi: 10.3389/fphar.2025.1726254 (PMC12819326; doi:10.3389/fphar.2025.1726254)
Supplement: Supplementary file 2 [file Supplementaryfile1.docx]

Supplementary Material

# Supplementary Tables

Table1. The sequence information of siRNA or overexpression plasmid.

| Gen name | Sequence | |
| --- | --- | --- |
|  | sense（5'-3'） | antisense（5'-3'） |
| NC | UUCUCCGAACGUGUCACGUTT | ACGUGACACGUUCGGAGAATT |
| siRNA-PI3K-1#（829） | GGAUCAAGUUGUCAAAGAAGA | UUCUUUGACAACUUGAUCCUG |
| siRNA-PI3K-2#（45） | GGCAAAUUAAACACCUUAAAU | UUAAGGUGUUUAAUUUGCCUG |
| siRNA-PI3K-3#（653） | GGAGGAAAUAACAAAUUAAUC | UUAAUUUGUUAUUUCCUCCUU |
| siRNA-AKT-1#（413） | GCUACUUCCUCCUCAAGAACG | UUCUUGAGGAGGAAGUAGCGU |
| siRNA-AKT-2#（824） | GGAAGGUGAUUCUGGUGAAAG | UUCACCAGAAUCACCUUCCCA |
| siRNA-AKT-3#（333） | GGAUACCAUGAACGACGUAGC | UACGUCGUUCAUGGUAUCCGU |
| pcDNA3.1-PI3K | >NM_001024955.2 Mus musculus phosphoinositide-3-kinase regulatory subunit 1 (Pik3r1), transcript variant 1, Mrna CATGTACACCACGGTTTGGACTATGGAAGACCTGGACTTAGAGTGTGCCAAGACAGATATAAACTGTGGCACAGACTTGGTGTTTTATATAGAAATGGATCCACCAGCACTGCCCCCCAAACCACCCAAGCCCACTACTGTAGCCAACAACAGCATGAACAACAATATGTCCTTGCAGGATGCTGAATGGTACTGGGGAGACATCTCAAGGGAAGAAGTGAATGAAAAACTCCGAGACACTGCTGATGGGACCTTTTTGGTACGAGACGCATCTACTAAAATGCACGGCGATTACACTCTTACACTAAGGAAAGGAGGAAATAACAAATTAATCAAAATCTTTCACCGTGATGGAAAATATGGCTTCTCTGATCCATTAACCTTCAACTCTGTGGTTGAGTTAATAAACCACTACCGGAATGAGTCTTTAGCTCAGTACAACCCCAAGCTGGATGTGAAGTTGCTCTACCCAGTGTCCAAATACCAGCAGGATCAAGTTGTCAAAGAAGATAATATTGAAGCTGTAGGGAAAAAATTACATGAATATAATACTCAATTTCAAGAAAAAAGTCGGGAATATGATAGATTATATGAGGAGTACACCCGTACTTCCCAGGAAATCCAAATGAAAAGAACGGCTATCGAAGCATTTAATGAAACCATAAAAATATTTGAAGAACAATGCCAAACCCAGGAGCGGTACAGCAAAGAATACATAGAGAAGTTTAAACGCGAAGGCAACGAGAAAGAAATTCAAAGGATTATGCATAACCATGATAAGCTGAAGTCGCGTATCAGTGAGATCATTGACAGTAGGAGGAGGTTGGAAGAAGACTTGAAGAAGCAGGCAGCTGAGTACCGAGAGATCGACAAACGCATGAACAGTATTAAGCCGGACCTCATCCAGTTGAGAAAGACAAGAGACCAATACTTGATGTGGCTGACGCAGAAAGGTGTGCGGCAGAAGAAGCTGAACGAGTGGCTGGGGAATGAAAATACCGAAGATCAATACTCCCTGGTAGAAGATGATGAGGATTTGCCCCACCATGACGAGAAGACGTGGAATGTCGGGAGCAGCAACCGAAACAAAGCGGAGAACCTATTGCGAGGGAAGCGAGACGGCACTTTCCTTGTCCGGGAGAGCAGTAAGCAGGGCTGCTATGCCTGCTCCGTAGTGGTAGACGGCGAAGTCAAGCATTGCGTCATTAACAAGACTGCCACCGGCTATGGCTTTGCCGAGCCCTACAACCTGTACAGCTCCCTGAAGGAGCTGGTGCTACATTATCAACACACCTCCCTCGTGCAGCACAATGACTCCCTCAATGTCACACTAGCATACCCAGTATATGCACAACAGAGGCGATGA |  |
| pcDNA3.1-AKT | >NM_001165894.2 Mus musculus thymoma  viral proto-oncogene 1 (Akt1), transcript variant 2, mRNA ATGAACGACGTAGCCATTGTGAAGGAGGGCTGGCTGCACAAACGAGGGGAATATATTAAAACCTGGCGGCCACGCTACTTCCTCCTCAAGAACGATGGCACCTTTATTGGCTACAAGGAACGGCCTCAGGATGTGGATCAGCGAGAGTCCCCACTCAACAACTTCTCAGTGGCACAATGCCAGCTGATGAAGACAGAGCGGCCAAGGCCCAACACCTTTATCATCCGCTGCCTGCAGTGGACCACAGTCATTGAGCGCACCTTCCATGTGGAAACGCCTGAGGAGCGGGAAGAATGGGCCACCGCCATTCAGACTGTGGCAGATGGACTCAAGAGGCAGGAAGAAGAGACGATGGACTTCCGATCAGGCTCACCCAGTGACAACTCAGGGGCTGAAGAGATGGAGGTGTCCCTGGCCAAGCCCAAGCACCGTGTGACCATGAACGAGTTTGAGTACCTGAAGCTACTGGGCAAGGGCACCTTTGGGAAGGTGATTCTGGTGAAAGAGAAGGCCACAGGCCGCTACTATGCCATGAAGATCCTCAAGAAGGAGGTCATCGTCGCCAAGGATGAGGTTGCCCACACGCTTACTGAGAACCGTGTCCTGCAGAACTCTAGGCATCCCTTCCTTACGGCCCTCAAGTACTCATTCCAGACCCACGACCGCCTCTGCTTTGTCATGGAGTATGCCAACGGGGGCGAGCTCTTCTTCCACCTGTCTCGAGAGCGTGTGTTCTCCGAGGACCGGGCCCGCTTCTATGGTGCGGAGATTGTGTCTGCCCTGGACTACTTGCACTCCGAGAAGAACGTGGTGTACCGGGACCTGAAGCTGGAGAACCTCATGCTGGACAAGGACGGGCACATCAAGATAACGGACTTCGGGCTGTGCAAGGAGGGGATCAAGGACGGTGCCACTATGAAGACATTCTGCGGAACGCCGGAGTACCTGGCCCCTGAGGTGCTGGAGGACAACGACTACGGCCGTGCAGTGGACTGGTGGGGGCTGGGCGTGGTCATGTACGAGATGATGTGTGGCCGCCTGCCCTTCTACAACCAGGACCACGAGAAGCTGTTCGAGCTGATCCTCATGGAGGAGATCCGCTTCCCGCGCACACTCGGCCCTGAGGCCAAGTCCCTGCTCTCCGGGCTGCTCAAGAAGGACCCTACACAGAGGCTCGGTGGGGGCTCCGAGGATGCCAAGGAGATCATGCAGCACCGGTTCTTTGCCAACATCGTGTGGCAGGATGTGTATGAGAAGAAGCTGAGCCCACCTTTCAAGCCCCAGGTCACCTCTGAGACTGACACCAGGTATTTCGATGAGGAGTTCACAGCTCAGATGATCACCATCACGCCGCCTGATCAAGTTCTCCTACTCAGCCAGTGGCACAGCCTGAGGCCTGGGGCAGCGGCTGGCAGCTCCACGCTCCTCTGCATTGCCGAGTCCAGAAGCCCCGCATGGATCATCTGA |  |

Table2 Primer Sequences

| Gene | Amplicon Size  （bp） | Forward primer  （5'→3'） | Reverse primer  （5'→3'） |
| --- | --- | --- | --- |
| β-actin | 120 | AGTGTGACGTTGACATCCGT | TGCTAGGAGCCAGAGCAGTA |
| AKT | 172 | CCAGGGGAGGATGTTTCTAC | GGTCGCGTCAGTCCTTAATA |
| NLRP3 | 176 | GCTGCTATCTGGAGGAACTT | TGAGGTCCACATCTTCAAGG |
| RIPK3 | 146 | GATTGTTCCCTTTGCAGACC | CAGAGGAACCGCATAACTTG |
| Caspase-9 | 177 | AATAAATCTTCGGCAATAGG | AGGAGACTTGATCTGTGGG |

Table 3 Information on Relevant Antibodies

| Product Name | Manufacturer | Item Number | Batch number | Molecular weight |
| --- | --- | --- | --- | --- |
| Caspase-1 | CST | 24232S | 5 | 48 kDa |
| Caspase-3 | abcam | ab184787 | GR3396760-7 | 32kDa |
| Caspase-8 | Bioworld | BS61777 | CN44171 | 57kDa |
| GSDMD-N | abcam | ab215203 | GR305773-2 | 31 kDa |
| PI3K | abcam | ab86714 | GR199664-6 | 84kDa |
| P-PI3K | abcam | ab182651 | GR305773-2 | 84KDa |
| AKT | CST | 4691s | 6 | 60KDa |
| P-AKT | CST | 4060s | 28 | 60KDa |
| MLKL | Proteintech | 21066-1-AP | 00081206 | 54KDa |
| p-MLKL | Proteintech | 82090-2-RR | 00010481 | 54KDa |
| GAPDH | Zsbio | TA-08 | 230040220 | 36 kDa |
